# Inpatient violence in a psychiatric hospital in the middle of the pandemic: clinical and community health aspects

**DOI:** 10.3934/publichealth.2022024

**Published:** 2022-02-22

**Authors:** Val Bellman, David Thai, Anisha Chinthalapally, Nina Russell, Shazia Saleem

**Affiliations:** 1 UMKC, Department of Psychiatry, Kansas City, MO; 2 Kansas City University of Medicine and Biosciences, Kansas City, MO, USA; 3 UMKC School of Medicine, Kansas City, MO; 4 University Health - Truman Medical Center, Kansas City, MO, USA

**Keywords:** violence, psychosis, pandemic, aggression, treatment, involuntary hospitalization

## Abstract

Healthcare workers are at a high risk of violence all over the world. The hostility toward nurses, physicians, and hospital staff has reached the point that it can be considered a public health problem. In this paper, we focus on the harassment, aggression, and violence that many healthcare workers have encountered while treating unstable psychiatric patients in the middle of the COVID-19 pandemic. We present a case with a history of violence toward mental health workers, review psychopathological and clinical aspects, and discuss how both the COVID-19 pandemic and current challenges in psychiatric hospital settings increase the frequency and severity of these attacks and how this affects the team on inpatient psychiatric units. We used the CARE guidelines to provide the most accurate and transparent information about the patient and relevant psychosocial aspects. We also pooled more than 20 unique sources to cover all aspects of violent behaviors in all psychiatric settings for all age groups. We concluded that a lack of nursing staff, the mental burden imposed by difficult patients, and poor communication between team members are some of the factors contributing to patient violence. An incomplete understanding of the problem creates barriers to change on both personal and systematic levels. Constant violence and abuse against healthcare workers cause stress, decreased productivity, and work dissatisfaction. To improve the safety of healthcare professionals, especially in inpatient psychiatric settings, several system-based changes should be implemented.

## 1. Introduction

Physical aggression and violence against healthcare workers is a serious problem that has been increasing over the past several years. Recent studies have elucidated how serious and commonplace these events can be in the middle of the pandemic [1]. In November 2020, National Nurses United (NNU) surveyed 15,000 nurses across the country and concluded that 20% of respondents reported increased workplace violence during the global pandemic. Moreover, 31% of nurses stated that workplace violence had increased even further, as reported in NNU's COVID-19 survey published in September 2021 [2]. Recent data published by the University of California, Berkeley's Human Rights Center revealed more than 1100 threats or episodes of violence against healthcare workers worldwide, while 400 incidents were directly related to the COVID-19 pandemic [3]. According to the CDC, almost 25% of healthcare workers report feeling bullied, threatened, or harassed because of the nature of their work and ongoing professional responsibilities [1]. In the context of COVID-19, rates of referral to mental health and psychological services have decreased, but the global pandemic has dramatically cut the availability of inpatient psychiatric beds, despite an increase in psychological distress and violence on inpatient units. The continued rise of workplace violence has been noted in mental health institutions and inpatient psychiatric settings across the country. In Missouri, a tripling of physical assaults against healthcare workers forced Cox Medical Center to order panic buttons for hospital staff to provide some measure of workplace violence prevention during the pandemic. In 2020, the number of episodes (including physical aggression and assaults) increased from 40 to 123, while reported injuries increased from 17 to 78. Although official numbers are still unavailable, hospital officials have reported that many of the episodes were directly related to underlying mental illness and/or substance use [4],[5]. Even before the pandemic, experts had grown increasingly worried about the problem of violence toward healthcare employees, including mental health workers, in Missouri-based hospitals [4],[6]. According to the Missouri Nurses Association, hostility and violence against healthcare workers increased dramatically from 2018 to 2020 [7]. The Kansas Hospital Association also emphasized that workplace violence in the Kansas/Missouri area was associated with an ongoing opioid crisis, substance abuse, psychiatric and behavioral problems, and staff shortages [8]. While their study did not focus on workplace violence in behavioral health facilities, the authors specifically outlined the inpatient psychiatric setting as “a risk factor for workplace violence.” The following case clearly demonstrates that unstable psychiatric patients may be a danger to themselves or others on the unit while indirectly contributing to staff shortages, as they are unable to understand the effects or the results of these violent actions.

## 2. Case presentation

### 2.1. Patient information

Our patient was a 30-year-old homeless, unemployed, single female with a past psychiatric history of bipolar I disorder, paranoid schizophrenia, PTSD, and generalized anxiety disorder; multiple psychiatric hospitalizations in three states; and unclear trauma history who was admitted to our inpatient psychiatric unit on an external 96-hour hold from jail due to violent behaviors and self-reported suicidal ideations, with a plan to strike her head against the wall. The toxicology screen was negative. She demonstrated lifetime patterns of insecurity, antisocial traits, impulse control problems, possessiveness in relationships, use of violence for need gratification, and an overall sense of inadequacy. Specifically, she reported ongoing thoughts of harming her half-brother and his significant other, and had at one point destroyed some of her half-brother's property. This patient also had a history of violent behaviors in jail, with several intimates being harmed or injured. The patient also had a lifelong pattern of faulty problem-solving skills, at least three past suicide attempts, and chronic substance abuse that led to impulse control problems and violent behaviors in three different treatment settings over the past five years. She tried multiple medications, including six antipsychotics, two mood stabilizers, and three anxiolytics, all with questionable compliance and poor treatment responses.

### 2.2. Clinical findings

During our initial encounter, the patient was noted to be pleasant and cooperative. Soon after, the patient began whispering to herself due to “physical pain”, referring to a car accident that had happened four days prior. She began exhibiting signs of acute mania and psychosis and was observed responding to internal stimuli. She verbally threatened resident physicians and left the room abruptly, which terminated the initial interview. Later, the patient gestured for the interviewers to return to her room. She handed them a piece of paper that was handwritten and split into boxes. She stated that the information provided was “patented” and warned providers not to copy it. Her sheet contained medications, goals (including being elected president by 2030), and different bits of her health and psychiatric histories.

### 2.3. Hospital course

The patient demonstrated a repeated pattern of threatening, harassing, intimidating, and bullying peers and staff members. During regular daily follow-ups with psychiatry residents, she would scream, curse, and use verbally abusive language when frustrated or stressed. She made excessive demands and unreasonable requests that needed to be met instantly. She used intimidating behavior to influence others to act in ways that tended to satisfy her personal needs, and failed to accept responsibility for deceitful behavior. She continued to threaten other patients on a daily basis and eventually required manual hold and mechanical restraints. There was an escalation of physical violence toward patients and staff, including throwing water, punching, and attacking others, including two psychiatry residents. Nursing staff consistently needed to move other patients from one unit to another due to safety concerns. Other patients also repeatedly questioned the staff's ability to handle this situation. The presence of this patient and the ongoing violence on the unit influenced the job performance, recruitment, and overall professional quality of life of residents and nursing staff. At least two team members reported that they were “extremely concerned” about issues including their safety around the patient, dysfunctional team dynamics and ineffective management of behavioral emergencies. Although specific numbers are not known, some staff members were either considering or planning to leave their current position because of dangerous staffing practices and being under too much pressure. Specifically, some members of our nursing team struggled to feel safe when attempting to respond to a patient's needs and were ready to quit or call in sick due to ongoing safety concerns. Already brutal shifts could easily turn into “extra stress” as other staff members walked away from the pressure, forcing other healthcare workers to stay with the patient and monitor for any changes in her behaviors. Additionally, staff themselves occasionally needed to be moved from one unit to another, and questioned the reasons for this patient's hospitalization, especially during the time of high acuity on the units. That, combined with the realization that some healthcare team members could make the same amount of money — if not more — on unemployment. Many of our team members, including one psychiatry resident, ended up needing to go to the emergency department for evaluation of their physical injuries. The patient was then placed on a 21-day involuntary hold for psychiatric stabilization.

Despite partial improvement, she continued to act impulsively. We petitioned for a 90-day involuntary commitment, but the patient eventually consented to the extended hospitalization. Staff had to move this patient to an adjacent unit due to her physically attacking a peer after a long period of no physically violent behavior. Again, this patient continued exhibiting violent behaviors, such as throwing liquids at others, becoming verbally agitated while saying somewhat unclear or nonsensical statements, and showcasing increasing emotional lability. By the end of her extended hospitalization, she was able to interact consistently with residents, staff members, authority figures, and peers in a nonthreatening manner.

### 2.4. Diagnostic assessment

Diagnostically, this patient met the criteria for schizoaffective disorder, bipolar type, as she remained manic and actively homicidal toward others for three weeks. Moreover, this diagnosis was confirmed by other reports and collateral information received prior to the patient's discharge. The patient also demonstrated a clinically significant problem in personality functioning that did not fit into any of the other existing personality disorder categories. She was diagnosed with unspecified personality disorder in accordance with DSM-5 criteria.

### 2.5. Therapeutic interventions

Our team started her on paliperidone 6 mg daily for mania and psychosis. Additionally, PO olanzapine 10 mg q2h PRN and IM olanzapine 10 mg q2h PRN were chosen for agitation. PO chlorpromazine 50 mg q4h PRN and IM chlorpromazine 25 mg q4h PRN were chosen for severe agitation, and if olanzapine was exhausted. She was finally stabilized on PO chlorpromazine at 100 mg QAM, 100 mg with lunch, and 200 mg with dinner for psychosis with IM back-up (chlorpromazine 25 mg); oxcarbazepine 600 mg QAM and 1200 mg QPM; paliperidone 12 mg daily; melatonin to 9 mg QHS; and trazodone 100 mg QHS. With these medication changes, our patient was still having recurrent verbal outbursts but eventually became less aggressive and violent. The treatment team and staff established and maintained appropriate boundaries and set firm, consistent limits when the patient had verbally or physically aggressive reactions.

### 2.6. Follow-up and outcomes

We were able to significantly reduce the frequency and intensity of angry verbal outbursts and assaultive/aggressive behaviors and terminate all acts of violence toward staff and residents. Ultimately, the patient was able to express anger through controlled, respectful verbalizations and healthy physical outlets on a consistent basis. There was a potential correlation between the amount of restful sleep the patient began getting and her outbursts the following day. She demonstrated marked improvement in terms of psychosis, mania, and the ability to listen and respond to the thoughts, feelings, and needs of others.

## 3. Discussion

**Figure 1.**
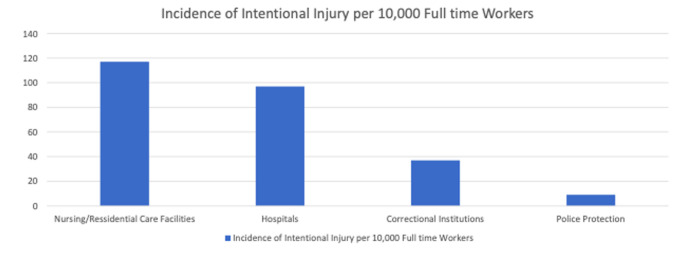
Incidence of intentional injuries in various settings. Adopted from: https://www.ashclinicalnews.org/spotlight/hazardous-health-violence-health-care-workplace/ [10].

Aggressive behavior and violent actions toward healthcare professionals have been well documented in many studies for the past 25 years. For example, Rosenthal [9] showed that 34.4% of healthcare workers had reported a physical assault within 12 months. More recently, a survey conducted by the Kansas Hospital Association in 2019 revealed that behavioral and substance use issues were the most important contributing factors to in-hospital violence and assault [8], indirectly confirming the increased risk of violence on inpatient psychiatric units. Healthcare settings have a higher incidence of assaults and intentional injuries than police stations or correctional facilities [10], while psychiatry-specific numbers are still unavailable. Figure 1 provides further details regarding intentional injuries in various settings.

Physical assaults represent a persistent problem in hospital psychiatry, with a range of 5% to 15% of the inpatient population committing physical assault [11]. More specifically, Kwok [12] showed in a review that psychiatric residents within cross-sectional studies have been found to have experienced at least one physical assault up to 64% of the time. Dvir [13] also showed that outside of physical assault, 86% of psychiatric residents had been previously threatened by a patient during their training. Residents may also have increased feelings of anxiety and depression, decreased job satisfaction, and increased thoughts of dropping out of residency [14].

### 3.1. Diagnostic and clinical aspects of aggressive behavior

Clinical and diagnostic factors also contribute to increased aggression. The clinical variables related to increased aggression in patients have been associated with certain disorders, such as psychotic disorders, personality disorders, and bipolar disorder, specifically regarding a manic state [15]. Fritz et al. [16] also suggested that individuals diagnosed with alcohol dependency, schizophrenia, or major depressive disorder possess higher self-aggressiveness when compared to healthy adults.

Bipolar disorder with comorbid antisocial personality disorder (ASPD) was previously described by Swann [17], who outlined that those who meet the criteria for both disorders were found to have higher rates of addictive, aggressive, and suicidal behaviors. Dunayevich [18] also noted that bipolar patients with certain personality traits have poorer outcomes in terms of potential recovery, behavioral instability, and relapse.

Schizophrenia with comorbid ASPD has been well described by several studies that concluded that men with both conditions showed tendencies toward violence [19],[20]. Moran et al. [20] showed that patients with schizophrenia or schizoaffective disorder and comorbid ASPD commit significantly more crimes than those with an individual diagnosis, and that they most commonly commit them before their first admission to psychiatric services. Schizophrenia and ASPD are individually associated with an increased risk of homicidality [21]. Similarly, diagnoses of bipolar disorder and cluster B personality traits were associated with increased patient impulsivity and hostility [22]. Although impulsiveness is not mentioned as a diagnostic criterion for schizoaffective or bipolar disorders, it is an important feature of several serious mental illnesses. In many cases, impulsivity can lead to other problematic behaviors, including violence, physical aggression, and self-harm.

### 3.2. Impact of the pandemic

**Figure 2.**
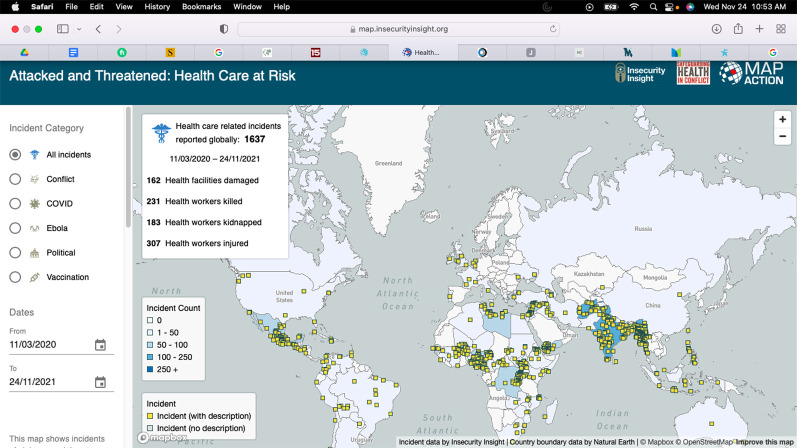
Attacked and threatened: health care at risk (11/03/2020–24/11/2021) [23].

While society, the media, and hospital officials have hailed healthcare workers as “heroes of the pandemic” for years, the US healthcare system has continued to neglect workplace violence prevention during the pandemic. Verbal and physical violence against healthcare workers is predominant, as these workers are the ones with the duty of endorsing masking and other restrictions due to the COVID-19 pandemic. Although healthcare workers experienced workplace violence before the pandemic began, the COVID-19 pandemic has tremendously exacerbated the problem. Figure 2 highlights workplace violence in healthcare across the world, as reported by Insecurity Insight [23].

Reportedly, many professionals are subject to health threats, bullying, and abuse, both on social media and in the real world. Payne-Gill et al. [24] confirmed that the COVID-19 pandemic resulted in a 35% increase in violent and aggressive cases due to local restrictions and poor patient–staff relationships. Interestingly, other authors noted significant reductions in aggression on inpatient psychiatric units during COVID-19 [25]. In some areas, there has been a dramatic rise in violence in psychiatric facilities and general hospitals [26],[27]. For example, in Long Island, New York, the main psychiatric hospital has seen a 60% rise in violence [28]. Part of this has been due to a 30% decrease in the pre-jail population, restricted access to long-term psychiatric care, and COVID-related court closures [28]. Interestingly, a very similar increase in patients' aggression was also found during the H1N1 pandemic in 2009 [25]. Most incidents of aggression occurred on acute admission or locked units, with a prevalence ranging from 44% to 62% on chronic units, and 30% in the outpatient department [15]. The increase in aggression in patients can be triggered not just by the patient's clinical presentation, but also by the nature of the clinical work, which involves coming in close contact, assessing, and interacting with patients on a daily basis [29]. Kinoshita et al. [30] also found that violent behavior prior to hospitalization increased the chances of earlier discharge, which could affect patients' recovery in the middle of the pandemic. Table 1 provides additional information regarding workplace violence obtained during a survey of Michigan nurses in June 2020 [31].

**Table 1.** Inpatient care setting by workplace violence (06/2020) [31].

Inpatient care setting	Estimated violence against RN & LPN
Assisted living facility	27.1%
Psychiatric facilit	57.1%
Correctional facilities and detention centers	55.8%
Home health care	25.6%
Hospice	40.7%
Hospital	44.7%
Nursing home or long-term care facilities	32.5%

Bed occupancy rates represent one of the reigning issues that mental health wards face during the pandemic, contributing to increased aggression among patients in 2020–2021. Research suggests that the net occupancy of beds was higher on days with aggressive incidents than on days without. An excess between 5% and 10% contributed to increased risk for aggression, with an excess greater than 10% showing the largest risk [15]. Additionally, there were reported findings of unhygienic surroundings, inadequate quality of food, unavailability of daily necessities, and a lack of privacy that contributed to aggression [15].

### 3.3. Other contributing factors

There is a continuing shortage of psychiatric beds across the nation, since many facilities were instructed to reduce their capacity to meet epidemiological requirements or even to repurpose or redistribute psychiatric beds in order to hospitalize more patients with COVID-19 [32]. Massachusetts lost approximately 300 psychiatric beds in less than a year [24], while New York had to repurpose a brand-new long-term psychiatric care facility to a field hospital [33]. If a patient is aggressive, then in this time of shortage, it is nearly impossible to find them a bed.

Many behavioral health units in several states were closed due to the financial challenges presented by the ongoing COVID-19 pandemic, meaning unstable psychiatric patients must wait in the emergency room for hours and sometimes days, tying up emergency resources that could be allocated to other patients [34]. Moreover, the rate of people seeking routine mental health care in emergency rooms increased across the country during the global pandemic [35]. Many patients still have to leave without being seen because psychiatric patients cannot be moved for a number of reasons (e.g., unpredictable behaviors, acute psychosis, suicidal and homicidal state of mind). These challenges are associated with increased aggression in the hospital setting [34].

Most psychiatric hospitals across the nation are overwhelmed with violent attacks on their staff, but the increased stress due to the current pandemic has escalated this beyond previously seen levels [36]. One reason for the rise in cases of violence and aggression from 2012–2016 (when a notable rise in violence began) is the understaffing of mental institutions, which led to the over-burdening of the current health workers who cannot provide the necessary help to every single patient [37]. Inadequate staffing also represents an issue contributing to increased aggression on psychiatric units during the pandemic. Not only did reduced staffing lead to more aggression, as per the reports of nurses, but wards with high rates of aggression were found to have higher levels of unqualified staff and temporary staff. The Missouri Department of Mental Health reported that 35% of registered nurse positions, 57% of licensed practical nurse positions, and 32% of entry-level mental health worker or tech positions are vacant [38].

Psychiatric patients who have been found incompetent to stand trial by the court, cannot be simply released. Instead, they are expected to be sent to a psychiatric facility and treated by multidisciplinary team until they are restored to competency. Although these individuals are committed by the courts, they cannot be admitted to the hospital due to staffing issues, overcrowding in psychiatric wards and local epidemiological restrictions. These patients tend to remain in the correctional facilities without appropriate care [38]. The situation is so devastating that Virginia State officials had to close five of eight state-sponsored adult psychiatric facilities to new admissions due to unprecedented staffing shortages noted in the middle of the pandemic. According to data published in July 2021, more than 100 state-employed mental health workers resigned in just two weeks and quickly received multiple job offers in the private healthcare network while contributing to understaffing and other challenges such as increased workloads on nursing staff, greater employee stress, and lower morale on state-run psychiatric units [39].

**Figure 3.**
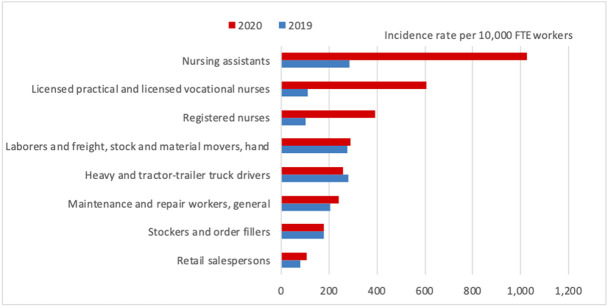
Incidence rates for cases resulting in days away from work in selected occupations, private industry, 2019–2020 [40].

According to the data provided by the Bureau of Labor Statistics, nursing assistants (including mental health techs), registered nurses, and licensed practical nurses had notable increases in their days away from work incidence rates in 2020 [40]. A very similar pattern was observed in the private industry, where nursing assistants also had a median number of 12 days “away from work,” which doubled since 2019 [40]. These numbers are outlined in Figure 3.

Issues concerning poor communication between staff and patients, a perceived lack of empathy, respect, and distance, or lack of shared decision making also contributed to increased aggression. Job strain associated with overwork, tiredness, job dissatisfaction, negative staff morale, and poor collaboration among nurses, as well as poor satisfaction with leadership, contribute to higher reports of aggression in the wards [15]. Dean et al. [41] reported that violence toward psychiatric nurses was one of the most common concerns for employers and staff. They highlighted the lack of support from the administration as another reason why health workers could not deal with violent patients.

Some private hospitals across the country tend to decline the most challenging psychiatric cases, while safety-net and community facilities have no choice but to admit these patients. This trend is expected to continue, raising questions about the impact that the policy will have on access to healthcare services in underserved communities.

There also exists an issue for mental health patients who are stuck in the emergency department for days on end, prolonging the psychiatric care they need. This experience in mental health care is so common that it has its own name: “boarding” which is defined as spending more than 12 hours in an emergency department waiting for an appropriate placement [42]. Since 2020, the number of patients who have boarded in emergency departments has increased by between 200% and 400% [42]. The importance of boarding being eradicated in mental health ward settings cannot be understated: in the long term, patients, especially children, with mental health issues who are not treated right away may deal with worsened symptoms and worsened aggression as a result of improper care.

### 3.4. Is there any room for improvement?

Though recommendations have been put in place by the American Psychiatric Association, WHO, and OSHA to increase training in managing patient violence, this is a persistent issue. A more recent review on violence against doctors by Kumari [43] commented on how it is potentially a more serious issue due to severe underreporting by physicians, possibly due to physicians not believing effective action could be taken if they were to report incidents [43]. Unfortunately, underreporting is also common among nurses all over the world [44],[45].

Medicare and Medicaid require community hospitals to administer and/or facilitate the Hospital Consumer Assessment of Healthcare Providers and Systems (HCAHPS) survey to measure patients' satisfaction with and perceptions of their hospital experiences [46]. Poor survey results could result in psychiatric hospitals forfeiting some reimbursements or negatively affecting federal funding. Moreover, the presence of inpatient psychiatric services is associated with lower HCAHPS scores and decreased reimbursement through the Centers for Medicare and Medicaid Services [47]. Healthcare workers tend to be afraid of HCAHPS results since low survey scores may negatively affect their paychecks and employment in general. It is also possible that hospital employees would be less likely to report occupational violence while indirectly contributing to the culture of silence [48]. Finally, this practice may unintentionally create an environment in which disrespectful and inappropriate behaviors are tolerated [49].

While mental health workers and professionals, especially those not in private practice, seem to be undercompensated compared to professions with similar educational requirements, the US healthcare system has failed to address this issue. Considering the significant safety risks, employees in mental health facilities should be compensated appropriately; oftentimes, they are not, leading to a lack of nursing staff and mental health technicians, as can be currently observed on state-run behavioral health units in Missouri [50].

Thankfully, state and hospital officials recognize the magnitude of the problem. At least two bills were introduced in 2020 with the goal of protecting hospital employees, motivating healthcare officials to develop workplace violence prevention plans (HB 398), and holding those who have assaulted healthcare workers accountable for their actions (HB 1022). According to local media, while the Missouri Nurses Association supports House Bill 398 for providing extra protection, violence prevention, and reporting opportunities, the Missouri Hospital Association does not. It remains unclear as to what factors contributed to this disagreement. Both professional organizations agreed that workplace violence is one of the reasons why we experience a shortage of professionals [51].

Various safety and security events in US healthcare facilities cost an estimated $1.6 billion each year. A recent analysis of hospital financial plans and recent spending in California shows that hospitals spend less than 0.5% of their total expenses on security, while the risks of violence are clearly underestimated [52]. According to a 2016 survey conducted by Health Facilities Management and the American Society for Healthcare Engineering, over 50% of hospitals had to increase their security budgets, but the results of those investments are still unclear [53]. Moreover, data from state-run or forensic facilities are not routinely published. Given the current circumstances, state-run and private healthcare networks need to spend more on security personnel and supporting staff. These simple efforts reduced violent crimes by 65% over 18 months in one New York-based hospital [54].

### 3.5. Ways to improve patient experience and staff safety in a psychiatric hospital

Lower aggression rates were found in hospitals that practiced positive teamwork, with staff feeling less pressured, having less of a workload, and feeling supported [15]. Yosep et al. [55] also concluded that labeling or ignoring the patients' coping style could lead to an increase in violent cases among patients with schizophrenia [55]. There should be higher staff-patient ratios in both the dayshift and the nightshift, as well as more dayroom space and bedroom space per patient. To improve comfort, some factors that can be changed include having a higher indoor temperature, having lower noise levels, having fewer rooms, including more single occupancy rooms, and providing the opportunity for patients to participate in games with others [55]. It has also been noted that wards that included exclusively green or rural scenery reported significantly fewer physical and verbal incidents than wards with industrial scenery. Rural views may be perceived as more pleasant and potentially more relaxing than viewing manmade structures [55]. Including more outdoor space has also been highly recommended to help bolster social interaction and recovery; however, issues like limited staff availability to escort patients to outdoor spaces may prevent this from happening [55].

In light of other issues related to the global pandemic, hospitals have been working to add more inpatient psychiatric beds; for example, in December 2020, the Cambridge Health Alliance added 42 beds for children with autism and other neurodevelopmental disorders [42]. Some hospitals have attempted to scale down and suspend outpatient and nonessential services to streamline resources to inpatients. There were also outdoor courtyards that were opened to allow for scheduled breaks, and patient engagement steering groups were established to promote engagement in inpatient units. Additionally, in April 2020, adolescent units were combined into one unit, and more patient and family engagement activities were organized to allow for more meaningful interactions [25]. Altogether, these measures are supposed to prevent further behavioral outbursts in this challenging population.

## 4. Conclusions

The COVID-19 pandemic has played a drastic role in the operation of US healthcare. We have seen that many mental health services offered by facilities have been indefinitely suspended, there have been patients in need of help having their care delayed, and we have seen a tremendous transition to virtual care. Although more studies are needed to establish a more definitive relationship between workplace violence in healthcare and mental health settings and pandemic-related factors, the majority of attacks come from patients who have problems with substance abuse and mental illness. Patient-related factors, such as homelessness and low socioeconomic status, and system-based aspects, such as staffing shortages, have been found to contribute to increased aggression in hospital settings. Ultimately, patient aggression is at its core a barrier to best practice and efficiency in care. It is a challenge that can delay treatment, placement, and stunt the best possible outcome a patient may have after a course of inpatient treatment. Although the clinician's right to refuse treatment of violent patients is allowed by law, many practitioners still treat all patients who need our help, regardless of their actions or abusive potential. Unfortunately, no matter what data are presented, medical and psychiatric facilities consistently stand out as some of the most dangerous workplaces in the United States. This article is expected to emphasize the paramount need for effective prevention of violence against healthcare and social service workers. Although many facilities have instituted measures against violence, there is much more work to be done.
